# Supplementary figures and images for: Molecular asymmetry in the cephalochordate embryo revealed by single-blastomere transcriptome profiling
Source: PLoS Genet. 2020 Dec 31;16(12):e1009294. doi: 10.1371/journal.pgen.1009294 (PMC7806126; doi:10.1371/journal.pgen.1009294)

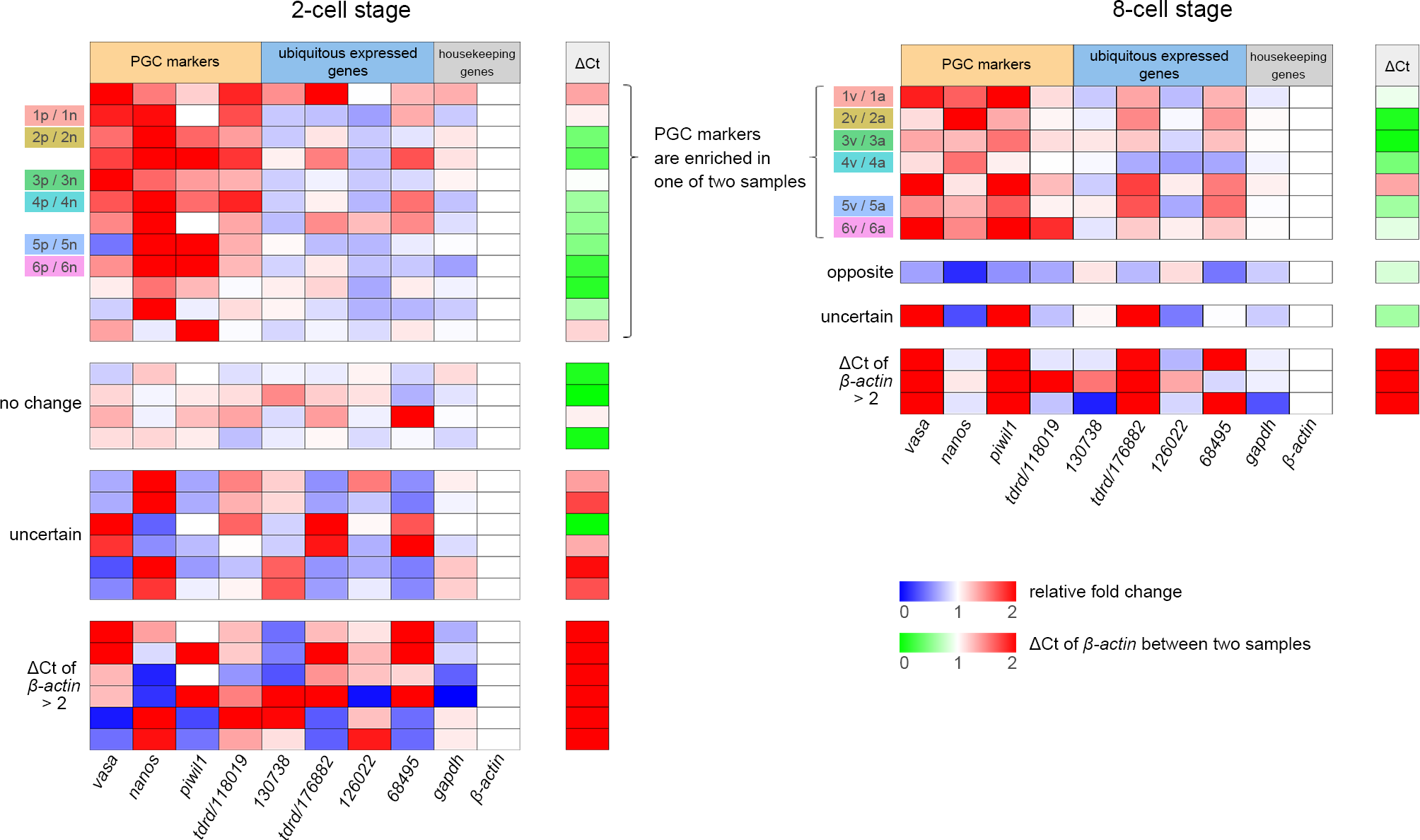

Supplement: S1 Fig — Heatmaps show the relative fold changes between two samples from the same embryo for the indicated transcripts. The relative fold changes were normalized to β-actin. The names of characterized transcripts or their IDs of the gene models are shown at the bottom of the corresponding column. The embryo sets selected for RNA sequencing are indicated on the left side of the corresponding rows with different colors. The “no change” group represents embryos in which significant enrichment of germ granule-enriched transcripts could not be detected between the two separated blastomeres. The “uncertain” group indicates that the fold changes of characterized transcripts did not show the expected pattern. The “ΔCt of β-actin > 2” group includes samples that show disparities in the amount of cDNA between samples isolated from the same embryo, suggesting that RNA quality of one of the two paired samples was low. The “opposite” group indicates that the animal and vegetal tiers distinguished by morphology displayed an opposite pattern for fold changes of characterized transcripts. The red to blue color scale indicates relative fold changes between two samples from the same embryo. The red to green color scale indicates ΔCt values of β-actin between two samples from the same embryo. The presumptive germ granule-positive (p), germ granule-negative (n), animal-tier (a), and vegetal-tier (v) blastomeres are indicated. (TIF) [file pgen.1009294.s001.tif]

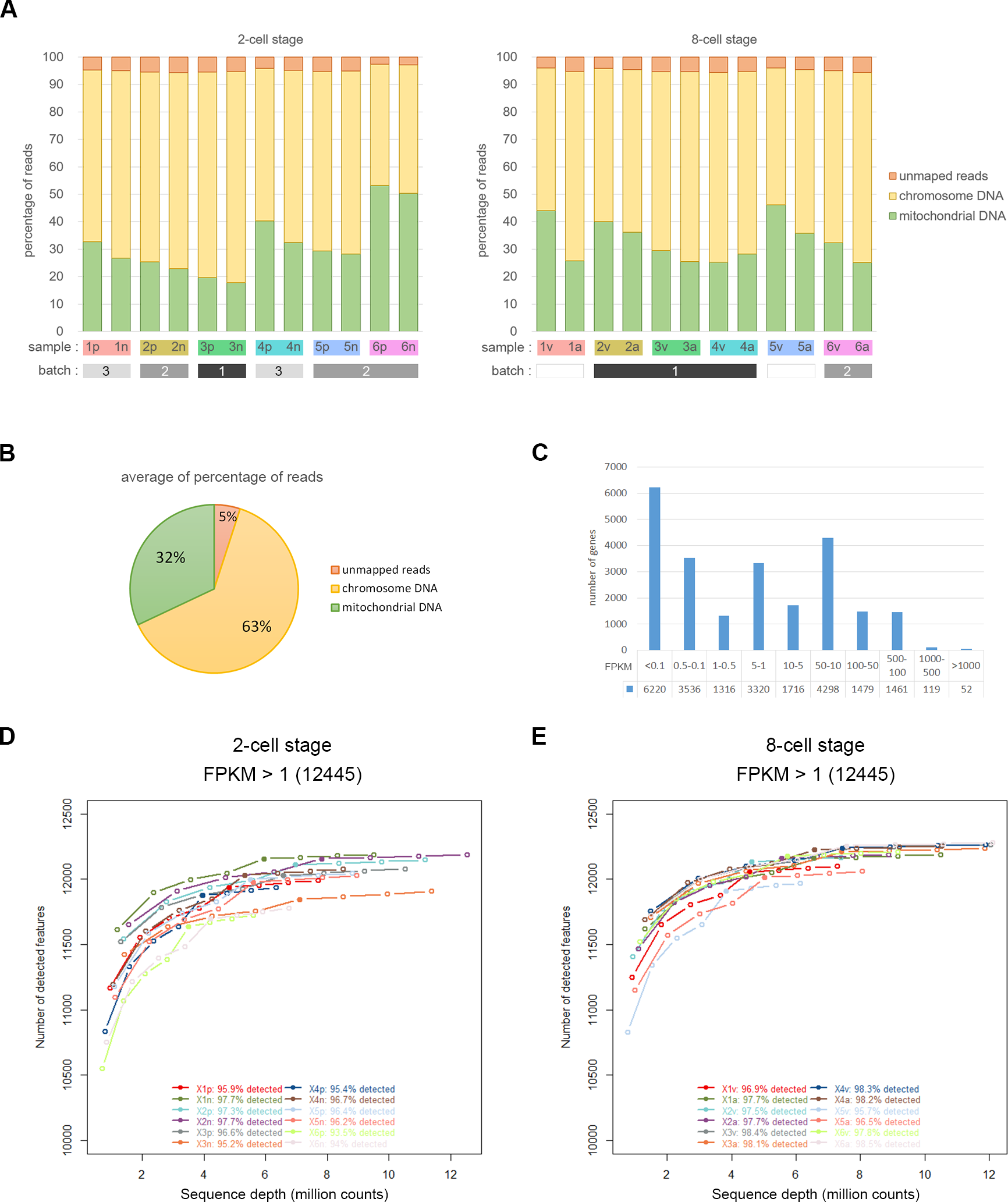

Supplement: S2 Fig — (A) Histograms showing the percentages of mapped and unmapped reads across twelve 2-cell stage or 8-cell stage samples. Identification of samples and their batch of origin are shown at the bottom of the histograms. (B) The pie chart shows the average ratios of mapped and unmapped reads. On average, 32% and 63% of reads were mapped to mitochondrial DNA (green) and chromosomal DNA (yellow), respectively, and 5% of reads could not be mapped (orange) to the reference sequence of B. floridae. (C) Distributions of average FPKM values for the 23,517 genes with detectable transcript levels. Among them, 12,445 genes showed moderate-to-high transcript levels (FPKM > 1). (D-E) Saturation curve analyses showed sequencing depths based on million counts over the number of genes with FPKM > 1 across 2-cell stage (D) and 8-cell stage (E) samples. Underlying data are available in S1 Data. (TIF) [file pgen.1009294.s002.tif]

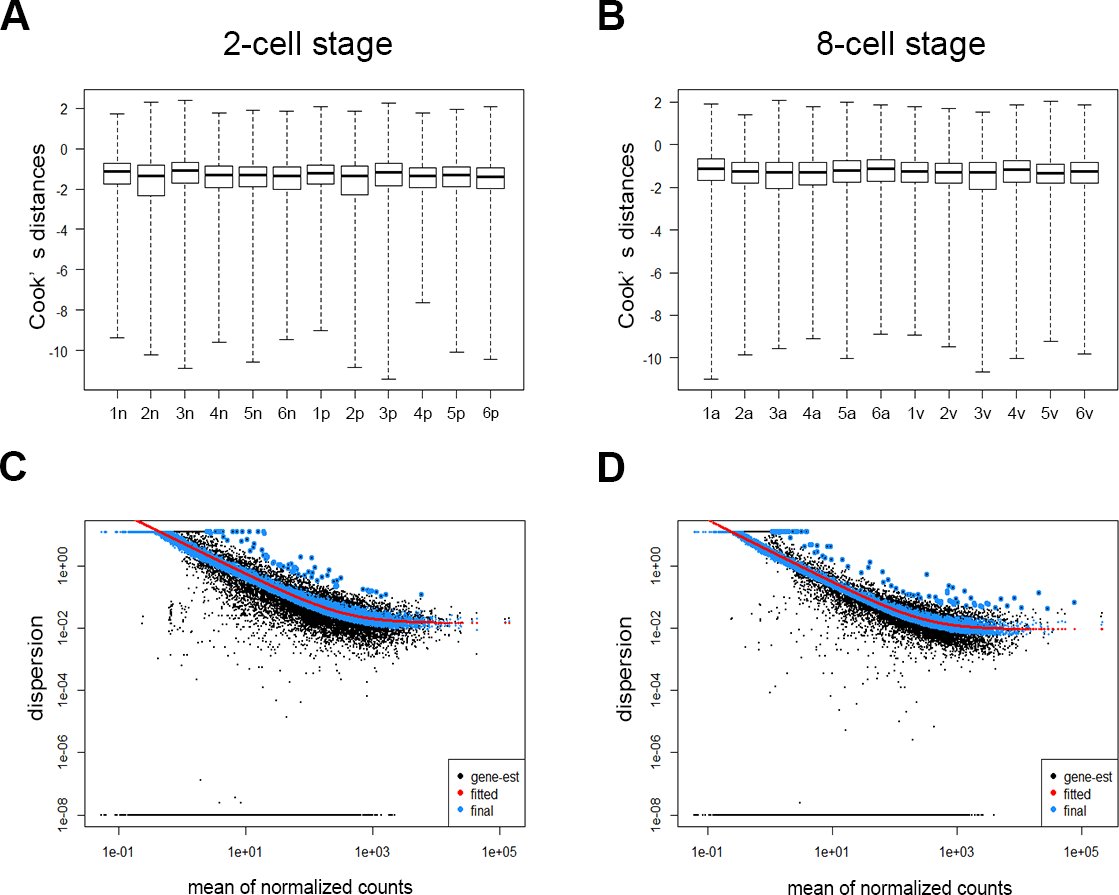

Supplement: S3 Fig — (A-B) Boxplot of Cook’s distances across the twelve 2-cell stage (A) and the twelve 8-cell stage (B) samples showed that there were no detectable outliers among samples. (C-D) The scatter plots show the estimated dispersion values over the mean of normalized counts for each gene in 2-cell stage (C) and 8-cell stage (D) samples. Black dots denote the dispersion values of each gene analyzed by maximum likelihood estimation (MLE) among the six biological replicates (12 samples). The red line denotes a common trend of dispersion for all samples. Blue dots denote final adjusted dispersion values of each gene analyzed by maximum a posteriori (MAP) estimation; the outliers are labeled with black dots surrounded by blue circles. (TIF) [file pgen.1009294.s003.tif]

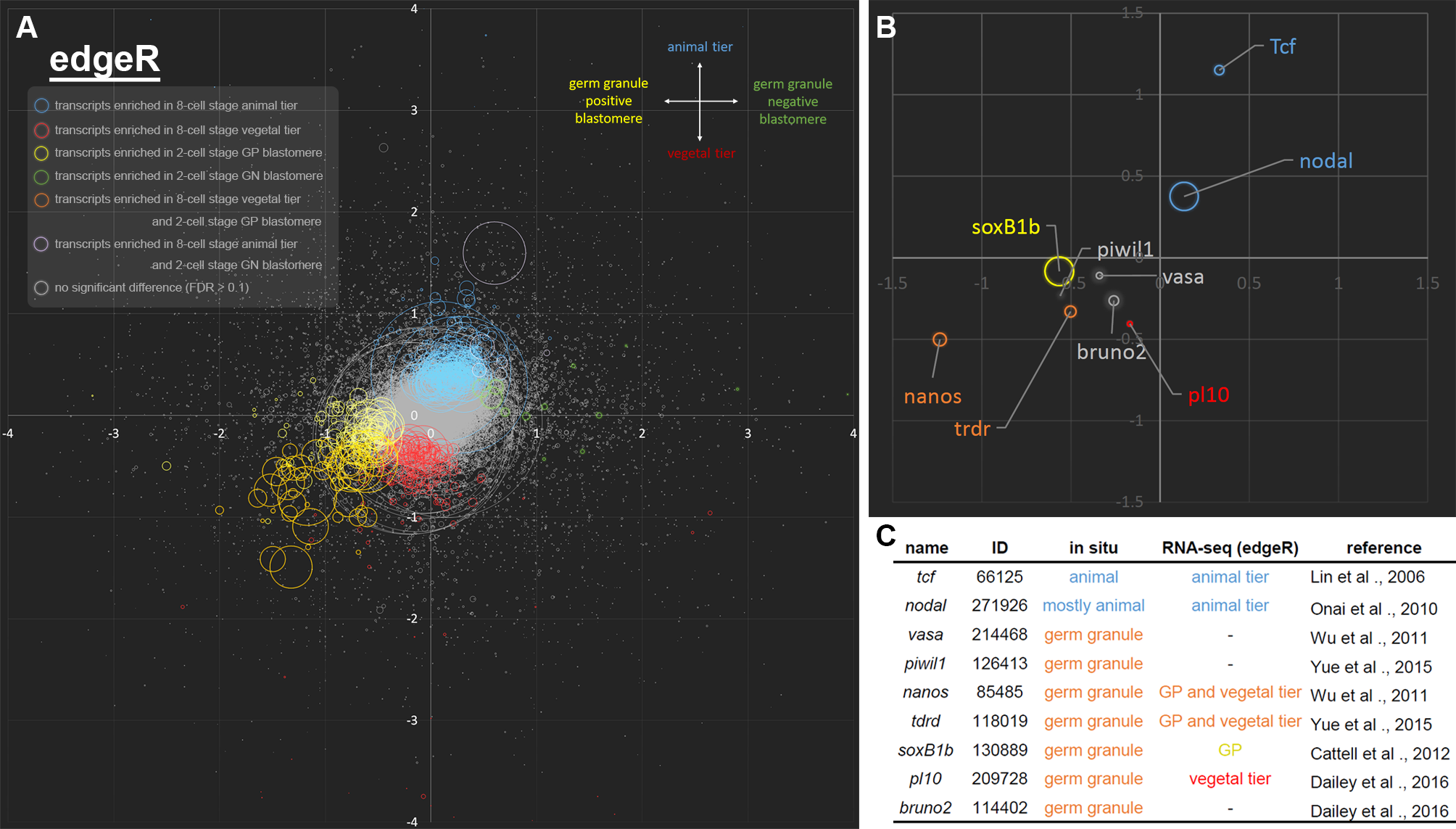

Supplement: S4 Fig — (A) All symbols are the same as those described in Fig 3. Blue circles (total number = 318) are DETs in the animal tier; red circles (188) are DETs in the vegetal tier; yellow circles (84) are DETs in germ granule-positive blastomere; green circles (15) are DETs in germ granule-negative blastomere; orange circles (60) are DETs in both the germ granule-positive blastomere and the vegetal tier; purple circles (5) are DETs in both the germ granule-negative blastomere and the animal tier. Transcripts that are not enriched are in gray. (B) A scatter plot showing the spatial distributions of nine previously characterized transcripts. (C) Summary information for the nine previously characterized genes. GP, germ granule-positive blastomere. ID, gene model ID from genome assembly v2.0. (TIF) [file pgen.1009294.s004.tif]

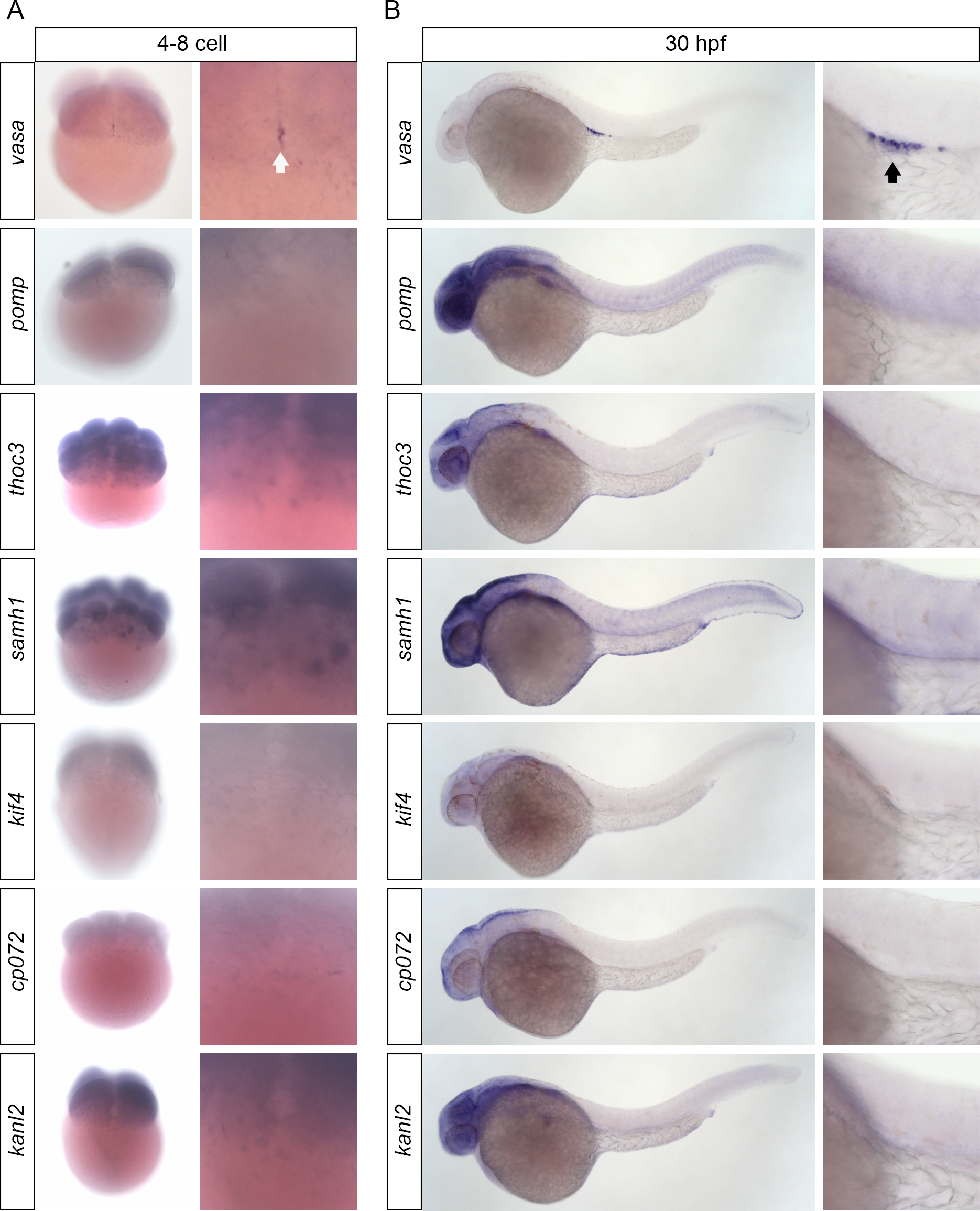

Supplement: S5 Fig — Whole-mount in situ hybridization of vasa, pomp, thoc3, samh1, kif4, cp072 and kanl2 in 4- to 8-cell stage (A) and 30 hpf (B) zebrafish embryos. The high-magnification views of the expected germ granule region (A) and developing gonad (B) are shown on the right-hand side of each panel. The arrows indicate signals of vasa transcripts. The data represent the expression patterns of most samples (>95%, n ≈ 30). (TIF) [file pgen.1009294.s005.tif]

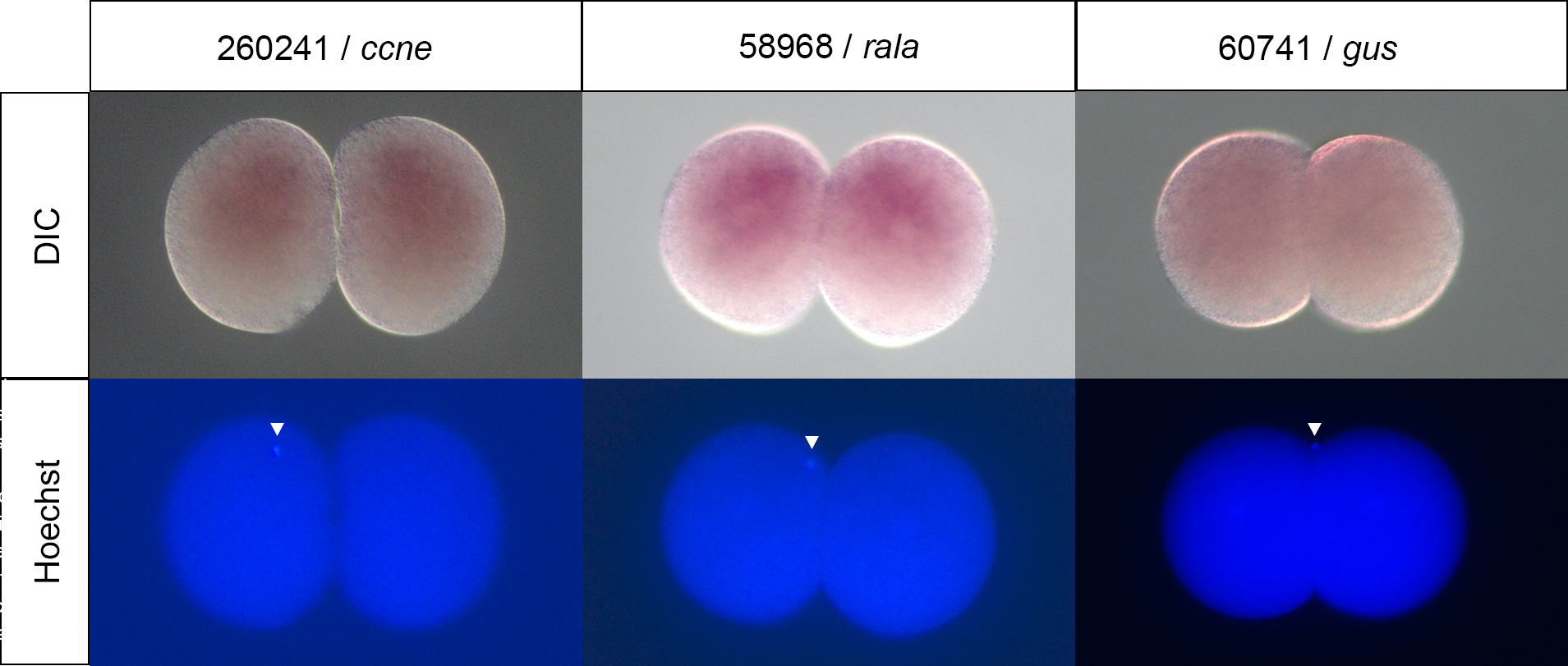

Supplement: S6 Fig — WMISH of three animal pole-localized transcripts at the 2-cell stage. The arrowheads indicate polar bodies visualized by DNA staining with Hoechst. (TIF) [file pgen.1009294.s006.tif]

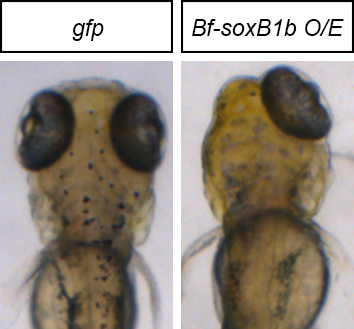

Supplement: S7 Fig — (TIF) [file pgen.1009294.s007.tif]

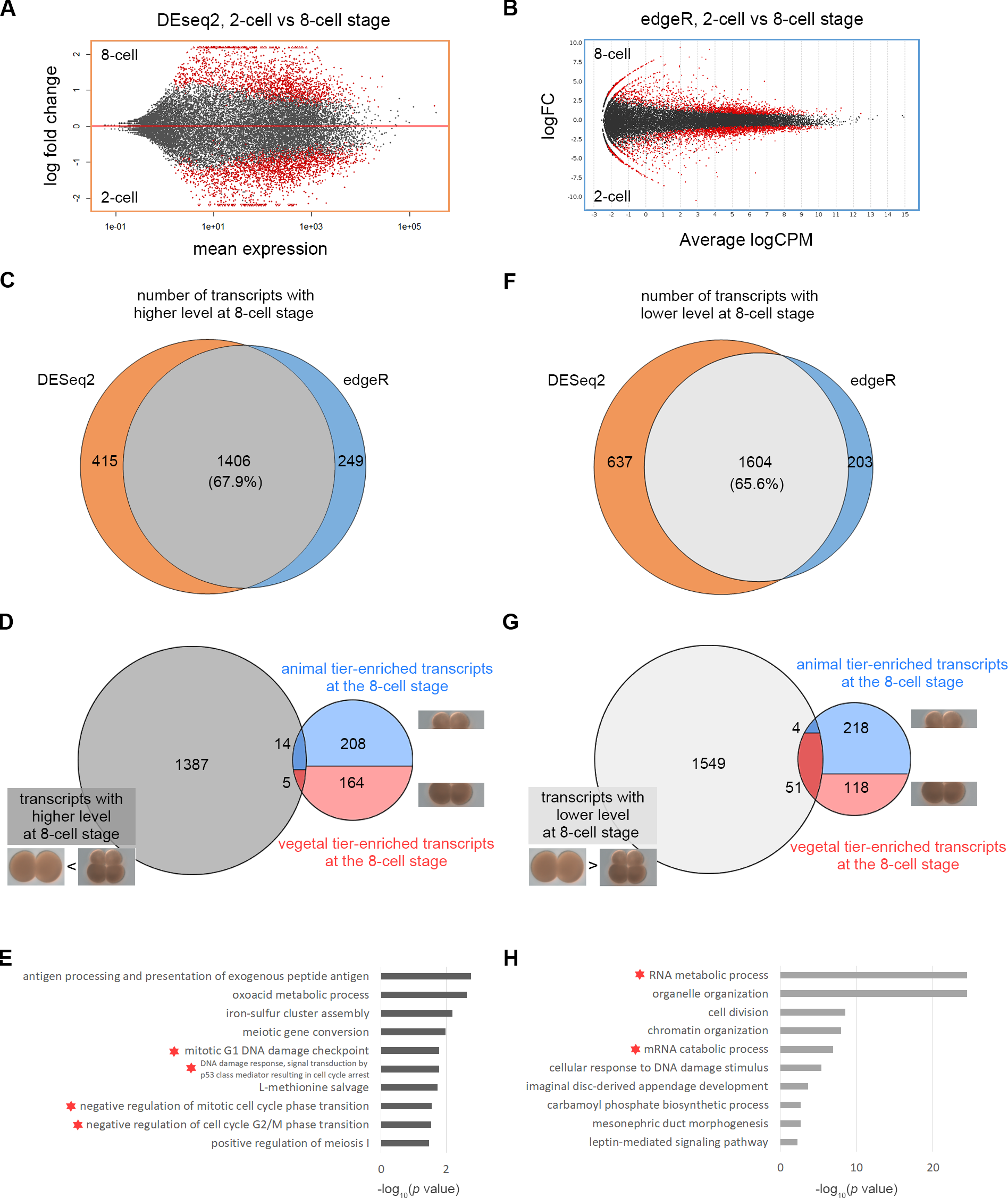

Supplement: S8 Fig — (A-B) MA-plots showing the log2 fold changes between the 2- and 8-cell stage embryos over the average transcript level of each gene by using DESeq2 (A) or edgeR (B). Transcripts showing significantly differential levels (FDR < 0.1) are in red. (C-D) Venn diagrams show the number of transcripts with higher level at the 8-cell stage (8-cell > 2-cell), comparing between DESeq2 and edgeR analyses (C), and the number of such transcripts with or without asymmetric localizations at the 8-cell stage (D). (E) Top 10 biological function GO groups of the transcripts showing higher levels at the 8-cell stage. (F-G) Venn diagrams show the number of transcripts with lower level at the 8-cell stage (8-cell < 2-cell), comparing between DESeq2 and edgeR analyses (F), and the number of such transcripts with or without asymmetric localization at the 8-cell stage (G). (H) Top 10 biological function GO groups of the transcripts showing lower levels at the 8-cell stage. For each group, the GO term with smallest p-value is shown. The asterisks indicate biological functions discussed in the text. Underlying data are available in S1 Data. (TIF) [file pgen.1009294.s008.tif]
