# Supplementary material for: Molecular asymmetry in the cephalochordate embryo revealed by single-blastomere transcriptome profiling
Source: PLoS Genet. 2020 Dec 31;16(12):e1009294. doi: 10.1371/journal.pgen.1009294 (PMC7806126; doi:10.1371/journal.pgen.1009294)
Supplement: S2 Data — (HTML) [file pgen.1009294.s012.html]

DESeq2


search 
   enter a JGI ID : 
Find   or
  
   
   select from list :   
   
GN/animal

↓ animal+GN ↓
125685 / p4r3a
198050 / zfp665

↓ GN only ↓
82748 / zn208
199243 / znt2
214534 / al4a1
265393 / s14l2

↓ animal only ↓
57072 / gch1
57204 / htai2
57405 / dus22
57510 / cant1
58190 / s19a3
58489 / pch2
58968 / rala
59314 / cc50a
59323 / gpc6
59448 / mmta2
59538 / spre
59930 / rad17
60741 / gus
61280 / surf4
61507 / kad3
63743 / t184c
63880 / dur3
63924 / ubp12
64320 / nol11
65694 / yipf4
65831 / tm180
66125 / tf7l2
67751 / cb076
68085 / pick1
68109 / nh2l1
68520 / sumo1
69231 / s14l5
69378 / zfp26
69715 / cc1
70265 / dag1
70370 / 
70379 / hypothetical protein BRAFLDRAFT\_70379
70406 / yipf1
70520 / klhl6
72535 / samc
72553 / 
72740 / kalm
73563 / acsf3
75840 / pabp1
76076 / ssrd
76421 / asm
76524 / pyrg1
79214 / hypothetical protein BRAFLDRAFT\_79214
79227 / hyls1
80438 / seltb
83499 / wun
83552 / cacp
83728 / cp39a
83775 / abh6b
83818 / hypothetical protein BRAFLDRAFT\_83818
84005 / ncln
84775 / ypc2
85926 / acsa
87680 / mtu1
87744 / t229b
87843 / pdk1a
87942 / mob2
88025 / pigq
89409 / rfa1
90748 / gld2
93596 / s39
95602 / pax3
95841 / acy2
96268 / pgfs
100087 / snx12
101561 / mftc
104997 / sumo1
105865 / fbln5
108787 / frmd6
113531 / xylk
114042 / acnt1
114107 / galt2
115231 / tmm98
115392 / nica
115435 / adpgk
115449 / erd22
115980 / clpt1
116025 / cp2u1
116259 / chm1b
117036 / zn706
117129 / aspg
117221 / spcs3
117512 / lfng
118255 / rbm18
118260 / cand1
118286 / mppa
118511 / s39a3
118530 / arrd3
118669 / gde1
118756 / 
118960 / CQ062
119014 / wipi3
119025 / retst
119264 / tean2
119286 / lerl1
119345 / tcta
119857 / pcsk7
120146 / itm2c
120194 / yhjx
120240 / CA198
120249 / cysk
120481 / rmd2
120531 / syt15
120761 / acbp4
120788 / bzw1
121170 / sia7b
121756 / fxl20
121867 / ubp32
121883 / b3glt
121900 / pmyt1
122172 / 
122501 / shcbb
122688 / pex14
122706 / erp44
122912 / mprd
123331 / sft2b
123482 / b3gl2
123522 / udb13
123860 / esc1
123924 / znt6b
124168 / sgmr1
124230 / eno4
124244 / mfs7a
124266 / b4gt7
124276 / gog7b
124326 / mp2k1
124328 / chss1
124584 / dck
124598 / skp1
124686 / aatc
124696 / 
124708 / cd63
124866 / spcs2
124957 / eif3i
124972 / sap3
125123 / kctd5
125407 / dox2
125408 / djc10
125474 / pkha2
125543 / armc1
125687 / 
125764 / swet1
125849 / pxdn
125975 / tmm60
125986 / dptor
126022 / mex3b
126377 / ppwd1
126485 / siat6
126702 / banp
126917 / mppe1
126922 / kith
126929 / ubc9
127441 / CT024
127845 / at2a1
127972 / tm241
128185 / ssrb
128825 / f154b
128934 / 
128972 / adt1
128977 / mtpn
129209 / pkhg1
129444 / gpr84
130184 / kbp
130938 / kcmb2
130962 / pmgt1
131048 / hdhd3
131390 / at135
131557 / pxl1
131972 / hypothetical protein BRAFLDRAFT\_131972
132165 / frmd6
141138 / rn185
141671 / srsf1
148820 / b3gt1
152479 / srsf3
176882 / akap1
179555 / pg12a
194453 / pkhg1
198184 / tx1b3
200264 / mp2k6
200281 / h2b1b
201921 / parl
202221 / syj2b
202693 / u2af4
203123 / prrt1
203577 / arhg3
204575 / scocb
205320 / cfc1
206753 / seh1
208190 / dhr11
208219 / ttll4
208404 / vang1
209836 / tm127
210496 / s39a9
211918 / litaf
212934 / ripb
213439 / klh24
215087 / vamp7
216392 / ppa6
217332 / siae
217512 / 
218680 / p3f4b
219228 / cdc45
219323 / a2mg
219600 / sia7b
220540 / aa
220927 / pdia4
226629 / lamc1
230000 / srsf7
230607 / pigb
231666 / fcl
231915 / ttc17
234358 / pabp1
234387 / btg3
237397 / asf1b
238322 / clp1l
239539 / diac
240864 / snx17
241140 / dhrs4
241182 / oaz1
241527 / mgat2
243769 / aktpa
247063 / mogs
250332 / sap
260130 / mfs11
260150 / osgi2
260241 / ccne
260671 / md2l1
261167 / arhgh
261267 / cyb5
261348 / c56d2
262109 / fut8
262959 / ap2s1
263424 / klh24
264353 / pyrd
268096 / ruxe
268308 / dyr
270549 / manf
270745 / met23
271926 / nodal
274883 / s5a1
274895 / aig1
275206 / nt5m
275883 / qpct
275989 / psug
276577 / sgcb
277055 / mfsd1
277118 / mspd1
277179 / s39ae
278662 / anky2
278774 / glrx3
278942 / pp2c5
279155 / a38
279229 / zn208
279446 / hypk
280392 / 
280636 / cp3ab
281090 / tm222
281522 / mknk1
281917 / bmt2
281938 / mest
282199 / tf2h2
284308 / catl
285628 / csn6
287844 / daw1
Find  
   
GP/vegetal

↓ GP+vegetal ↓
58704 / h2b7
61845 / ndk5
69591 / Y3380
73765 / klh24
74172 / hypothetical protein BRAFLDRAFT\_74172
83647 / nse1
85198 / dctn5
85485 / no1
86619 / mrp4
91042 / mlh1
92069 / lpd6b
104488 / rpc8
110219 / lpd6b
113678 / herc4
113894 / ubc
117694 / sycp3
118019 / stk31
118081 / hypothetical protein BRAFLDRAFT\_118081
118187 / glod5
118488 / kdm2b
118801 / rab6b
119023 / dnal4
120626 / cdn1b
121590 / nsapc
121868 / pop5
122454 / serb
122567 / ef1a
123295 / shrm2
124327 / rl17
124739 / kif4
124781 / tbx2
125125 / kif4
125204 / bambi
125219 / kbtb2
125397 / k1199
126143 / dep1b
126695 / samh1
128129 / thoc3
129676 / csn4
131234 / hypothetical protein BRAFLDRAFT\_131234
131415 / y381
147068 / kanl2
201484 / psa4
209238 / ddt4l
213143 / rm32
226282 / rt34
245871 / rt28
247418 / lsm3
247432 / foxn1
261932 / hebp2
267929 / pomp
268075 / psmd3
275420 / cp072
278100 / sfrp2
280755 / csn8
281075 / lsm4
283143 / rpab5

↓ vegetal only ↓
56819 / oxsm
57135 / metk2
57852 / strp1
58504 / dre2
60968 / morc2
63318 / camp1
63525 / hnmt
63857 / mtus2
63884 / ube3a
64995 / knop1
65029 / 
66480 / sp3aa
67212 / tdrkh
68217 / prp2
69036 / ush2a
69364 / qser1
69858 / hypothetical protein BRAFLDRAFT\_69858
70429 / nim1
72611 / zn208
73157 / syne1
75377 / zch18
75644 / hypothetical protein BRAFLDRAFT\_75644
76142 / myome
76211 / 
76434 / mltk
77377 / hypothetical protein BRAFLDRAFT\_77377
79014 / tripc
79692 / snx25
80092 / zn407
81739 / rs4
82314 / tob1a
82621 / chd1
82771 / t2fa
83265 / trim3
84515 / gblp
84681 / smrc2
84982 / epc1
85508 / pcgf3
85516 / ttf2
85597 / sac2
86023 / fkbp6
86166 / kif1c
86993 / hip1r
87954 / xfin
88457 / kbtb8
89765 / tyw3
90009 / ubp20
91830 / tchp
92256 / fat4
93483 / rfwd2
94947 / bag1
97793 / kbtb2
98696 / hypothetical protein BRAFLDRAFT\_98696
98697 / can15
99040 / swt1
100009 / ari4a
100648 / cac1b
101072 / st38l
102424 / grn
104092 / 
105111 / ttc40
105115 / simc1
105117 / exoc6
106805 / hirp3
113730 / rl22
113766 / atpb
114040 / brpf1
114402 / celf2
115078 / prs6b
115228 / ch60
115396 / smca1
115434 / ef1g
115608 / idhp
116232 / chtop
117612 / pur6
117750 / f194a
117769 / ccnb3
117888 / m4k2
118269 / rgl1
118301 / zn740
118356 / fzr
118418 / rl4b
118736 / fancm
118862 / cnot3
118868 / hypothetical protein BRAFLDRAFT\_118868
118871 / exos2
118942 / pfd6
118946 / rl7a
118991 / rbm7
119030 / l2gl2
119305 / tf3c1
119839 / h90a1
119860 / k1407
120339 / a15
120518 / vwa7
120897 / sulf1
121689 / zhx2
121698 / aggf1
121783 / 
122111 / ccd87
122755 / ddx17
122913 / f102a
123593 / ytdc2
123767 / ica69
123919 / fsip1
123925 / aqr
123988 / atf7
124063 / mocos
124134 / mprip
124240 / vkind
124260 / leucine-rich repeat-containing protein ddb
124602 / rt24
124709 / nek8
124790 / rbm19
124926 / abcf1
124989 / cxxc1
125124 / zn598
125154 / zc3h4
125196 / ptpr2
125520 / foxn4
125661 / nvl
126284 / prdm2
126355 / ccd17
126364 / lar
126427 / mifh
126580 / ep300
126921 / rs9
127756 / sp130
128224 / spt6h
128281 / lipa1
128562 / tep1
128687 / hypothetical protein BRAFLDRAFT\_128687
128832 / hells
129053 / ubc12
129638 / nsrp1
130062 / hypothetical protein BRAFLDRAFT\_130062
130762 / clspn
131119 / hypothetical protein BRAFLDRAFT\_131119
131216 / smc6
133792 / huwe1
133981 / kif19
135736 / ttc14
146334 / zn268
147013 / abt1
153828 / sbno1
153994 / apc
183747 / lrp4
183809 / cl16a
191392 / t10b
200232 / rrs1
201006 / kif15
201044 / rpa1
201563 / int6a
202569 / bub1b
204870 / nek1
205877 / YC006
206484 / rsf1
206850 / rasa1
208117 / afad
209377 / unc5c
209728 / ddx3y
210487 / mabp1
215173 / nuak1
215827 / af10
218963 / chd7
223882 / taf11
225211 / dph2
226459 / gptc4
232191 / dapk1
243011 / hdgr3
270524 / noc3l
270672 / serc
274292 / srp09
281016 / ttll1
281429 / morc2
283756 / msh6
285685 / rm45
286239 / rm43

↓ GP only ↓
58366 / gpn1
62075 / fggy
64101 / 64101
68564 / zn333
75729 / ninj1
78802 / ub2d4
86473 / nud18
86674 / rt35
87509 / ap4s
89899 / mcm8
89964 / hypothetical protein BRAFLDRAFT\_89964
93457 / nmrl1
109350 / cc28b
113733 / enoa
114609 / psb6
115265 / atg5
117094 / hypothetical protein BRAFLDRAFT\_117094
118403 / lsm7
118708 / ssa27
119142 / apc10
120483 / c1tc
120578 / dlgp1
121749 / comda
123617 / xrcc1
123635 / med11
123647 / cg055
123909 / macd2
124097 / atg10
124098 / rtp3
124137 / 42622
124144 / mid51
124436 / rm16
125393 / ap5s1
125988 / sir6
126025 / ubl7
126026 / cr3l4
127159 / hypothetical protein BRAFLDRAFT\_127159
127160 / hypothetical protein BRAFLDRAFT\_127160
127698 / iqcb1
127902 / md2l2
127903 / hypothetical protein BRAFLDRAFT\_127903
128014 / cd63
130314 / ing1
130889 / sox2
201261 / usb1
216798 / apop1
216834 / kcrm
217297 / med28
226533 / s39aa
231149 / ck049
235445 / kbtb2
239952 / atp5s
271452 / rm15
276010 / tbc19
277111 / wap53
277569 / nicn1
279056 / tcpb
287104 / ung
Find  
Clear
  
  
 
  
  

 information 

1. JGI ID :
2. symbol :
3. description :
4. FPKM :
5. FC(log2) -
6. X:
     
   Y:
