# Supplementary material for: Molecular asymmetry in the cephalochordate embryo revealed by single-blastomere transcriptome profiling
Source: PLoS Genet. 2020 Dec 31;16(12):e1009294. doi: 10.1371/journal.pgen.1009294 (PMC7806126; doi:10.1371/journal.pgen.1009294)
Supplement: S3 Data — (HTML) [file pgen.1009294.s013.html]

edgeR


search 
   enter a JGI ID : 
Find   or
  
   
   select from list :   
   
GN/animal

↓ animal+GN ↓
60741 / gus
123619 / abh6a
125685 / p4r3a
198050 / zfp665
208408 / hif1a

↓ GN only ↓
64349 / zg57
66499 / frm4a
71461 / cirbp
82716 / yr571
82748 / zn208
88006 / sem5a
124939 / glcne
128293 / 
199243 / znt2
210543 / pura2
228050 / klh21
265393 / s14l2
271947 / dyl1
279031 / rab28
287206 / syic

↓ animal only ↓
56698 / actm
57072 / gch1
57173 / dx39b
57204 / htai2
57405 / dus22
57510 / cant1
58190 / s19a3
58489 / pch2
58757 / dus10
58968 / rala
59276 / orml1
59314 / cc50a
59323 / gpc6
59448 / mmta2
59538 / spre
59803 / s35a2
59930 / rad17
60164 / facr1
60330 / ephx4
60348 / ggt1
60495 / zg57
61280 / surf4
61507 / kad3
61958 / usf2
63703 / cd033
63743 / t184c
63827 / glra1
63880 / dur3
63910 / pgm2
64320 / nol11
65483 / cab39
65694 / yipf4
65831 / tm180
66125 / tf7l2
66590 / nphp3
67039 / lpp1
67087 / p5cr1
67392 / cofi
67440 / svep1
67468 / c209e
67751 / cb076
68085 / pick1
68520 / sumo1
68918 / tm246
69231 / s14l5
69378 / zfp26
69484 / cept1
69715 / ccna1
70265 / dag1
70283 / sahh3
70370 / 
70379 / 
70406 / yipf1
70520 / klhl6
72535 / samc
72553 / 
72740 / kalm
73331 / nphp3
73563 / acsf3
75378 / ybet
75840 / pabp1
76076 / ssrd
76421 / asm
77926 / flot2
78425 / tm128
79214 / 
79227 / hyls1
80438 / seltb
80991 / 
82739 / b4gn1
82927 / gtr8
83499 / wun
83552 / cacp
83728 / cp39a
83775 / abh6b
83818 / 
84005 / ncln
84775 / ypc2
85123 / grid2
85450 / 
85926 / acsa
86427 / sar1b
86671 / phb1
87744 / t229b
87843 / pdk1a
87942 / mob2
88155 / ypc2
88981 / dpyl2
89407 / merl
90450 / yrdc
90640 / s17a5
90748 / gld2
90777 / mare3
92867 / sia4b
93596 / nas39
94253 / mgat1
94577 / notc2
95602 / pax3
95841 / acy2
96268 / pgfs
96403 / 
98873 / 
100087 / snx12
100695 / ppr32
101561 / mftc
102461 / fxl18
104997 / sumo1
105411 / ldlr
105600 / s39ac
105865 / fbln5
107299 / nb5r5
108787 / frmd6
108788 / 
113531 / xylk
113725 / ttc38
114042 / acnt1
114107 / galt2
115231 / tmm98
115392 / nica
115435 / adpgk
115449 / erd22
116025 / cp2u1
116259 / chm1b
116976 / kprs4
117129 / aspg
117148 / t184c
117220 / fry
117221 / spcs3
118260 / cand1
118286 / mppa
118511 / s39a3
118530 / arrd3
118669 / gde1
118756 / 
118960 / cq062
119014 / wipi3
119025 / retst
119345 / tcta
119739 / bivm
119857 / pcsk7
120079 / apx
120146 / itm2c
120194 / yhjx
120240 / ca198
120249 / cysk
120281 / fyco1
120481 / rmd2
120531 / syt15
120761 / acbp4
121756 / fxl20
121867 / ubp32
121873 / mmp16
121883 / b3glt
121900 / pmyt1
122172 / 
122237 / at1b1
122465 / aldr
122501 / shcbb
122688 / pex14
122747 / rnoy
122912 / mprd
123260 / 
123275 / aph1a
123331 / sft2b
123482 / b3gl2
123522 / udb13
123885 / cgat1
123924 / znt6b
123996 / ypc2
124168 / sgmr1
124230 / eno4
124244 / mfs7a
124266 / b4gt7
124276 / gog7b
124328 / chss1
124598 / skp1
124686 / aatc
124696 / 
124708 / cd63
124866 / spcs2
124957 / eif3i
124972 / sap3
125123 / kctd5
125408 / djc10
125474 / pkha2
125543 / armc1
125687 / 
125708 / ros1
125764 / swet1
125849 / pxdn
125975 / tmm60
126022 / mex3b
126152 / ykt6
126377 / ppwd1
126485 / siat6
126922 / kith
126929 / ubc9
127104 / 
127441 / ct024
127845 / at2a1
127972 / tm241
128102 / tor1b
128185 / ssrb
128825 / f154b
128934 / 
128972 / adt1
128977 / mtpn
129209 / pkhg1
129444 / gpr84
129636 / 
130184 / kbp
130938 / kcmb2
131048 / hdhd3
131557 / pxl1
132165 / frmd6
135372 / doxa1
141138 / rn185
141671 / srsf1
148820 / b3gt1
149126 / rab34
152479 / srsf3
176882 / akap1
179555 / pg12a
183960 / papog
194453 / pkhg1
198184 / tx1b3
199751 / tmc7
200264 / mp2k6
200281 / h2b1b
200350 / plbl1
201287 / mtfp1
201657 / tnf10
201921 / parl
202221 / syj2b
203123 / prrt1
203204 / cep41
203577 / arhg3
204575 / scocb
205320 / cfc1
206753 / seh1
208190 / dhr11
208219 / ttll4
210496 / s39a9
212546 / p5cr1
212934 / ripb
213439 / klh24
213467 / tx261
213876 / hyep
215087 / vamp7
215923 / asm
216392 / ppa6
217512 / 
218264 / pp4c
218680 / p3f4b
219228 / cdc45
219600 / sia7b
220540 / naaa
221786 / aldr
222452 / ticn2
224232 / nfyb
225597 / wdr31
226629 / lamc1
230000 / srsf7
231666 / fcl
231915 / ttc17
234358 / pabp1
234387 / btg3
237397 / asf1b
238322 / clp1l
239539 / diac
240864 / snx17
241140 / dhrs4
241182 / oaz1
241527 / mgat2
243769 / aktpa
247063 / mogs
254991 / mcu
260130 / mfs11
260150 / osgi2
260241 / ccne
261167 / arhgh
261267 / cyb5
261348 / c56d2
265104 / tsn7
267000 / m2om
268096 / ruxe
268308 / dyr
270549 / manf
271926 / nodal
274883 / s5a1
274895 / aig1
274964 / tri54
275206 / nt5m
275793 / tmx1
275883 / qpct
276577 / sgcb
277055 / mfsd1
277118 / mspd1
277179 / s39ae
278662 / anky2
278774 / glrx3
279155 / naa38
279229 / zn208
280392 / adx
280636 / cp3ab
281522 / mknk1
281532 / snx30
281917 / bmt2
281938 / mest
282199 / tf2h2
282701 / ccd53
283414 / 1433e
284104 / tm9s4
284308 / catl
285628 / csn6
286684 / ece1
287019 / plbl2
287844 / daw1
Find  
   
GP/vegetal

↓ GP+vegetal ↓
58704 / h2b7
61845 / ndk5
69591 / y3380
73765 / klh24
74172 / 
74957 / mrp4
83647 / nse1
85198 / dctn5
85485 / nano1
86619 / mrp4
91661 / dock7
92069 / lpd6b
104488 / rpc8
110219 / lpd6b
113678 / herc4
113894 / ubc
117694 / sycp3
118019 / stk31
118081 / 
118187 / glod5
118488 / kdm2b
118801 / rab6b
118991 / rbm7
119023 / dnal4
120518 / vwa7
120626 / cdn1b
121590 / nsapc
121783 / 
122454 / serb
122567 / ef1a
123295 / shrm2
123647 / cg055
124327 / rl17
124739 / kif4
124781 / tbx2
125397 / k1199
126143 / dep1b
126695 / samh1
127160 / 
127903 / 
128129 / thoc3
131234 / 
147068 / kanl2
201261 / usb1
209238 / ddt4l
213143 / rm32
216798 / apop1
226533 / s39aa
245871 / rt28
247418 / lsm3
247432 / foxn1/4b
261932 / hebp2
267929 / pomp
268075 / psmd3
275420 / cp072
278100 / sfrp2
280755 / csn8
281075 / lsm4
283143 / rpab5
286239 / rm43

↓ vegetal only ↓
56938 / top3a
57135 / metk2
57852 / strp1
58504 / dre2
60968 / morc2
62401 / apmap
63272 / flrt3
63318 / camp1
63624 / 
63857 / mtus2
65029 / 
66022 / zswm8
66480 / ycf45
67008 / trio
67212 / tdrkh
67491 / mint
67578 / nav3
68513 / 
69319 / jmjce
69364 / qser1
69858 / 
70225 / 
70429 / nim1
72611 / zn208
73140 / cd53
73157 / syne1
74200 / ttc28
75503 / trim2
75644 / 
76136 / 
76142 / myome
76168 / kdm8
76434 / mltk
76999 / ush2a
77679 / 
79014 / tripc
79692 / snx25
80922 / ank3
81739 / rs4
82621 / chd1
82678 / notch
83075 / spg17
83261 / dyh7
83779 / dop1
84473 / cenpe
84479 / dlg5
84515 / gblp
84653 / al2sa
84654 / zn436
84681 / smrc2
84982 / epc1
85508 / pcgf3
85516 / ttf2
85678 / tim
86166 / kif1c
86422 / klh21
86993 / hip1r
87954 / xfin
88457 / kbtb8
88587 / atm
90009 / ubp20
91624 / ecm29
91660 / dock7
92256 / fat4
93483 / rfwd2
93728 / 
94631 / hmgn5
95684 / nf1
95792 / iqga2
96053 / ard1
97793 / kbtb2
98696 / 
98697 / can15
99040 / swt1
100648 / cac1b
101072 / st38l
101777 / zn329
101973 / sia8b
104092 / 
105111 / ttc40
105115 / simc1
105117 / exoc6
106693 / iqec1
113730 / rl22
113850 / myh10
114040 / brpf1
114709 / glbl2
114976 / actm
115078 / prs6b
115228 / ch60
115396 / smca1
115608 / idhp
117750 / f194a
117888 / m4k2
118356 / fzr
118418 / rl4b
118736 / fancm
118862 / cnot3
118868 / 
118871 / exos2
118946 / rl7a
120136 / rpb1
120339 / naa15
120900 / rl19
121689 / zhx2
121698 / aggf1
121800 / utp20
122111 / ccd87
122755 / ddx17
123556 / fak1
123767 / ica69
123919 / fsip1
123925 / aqr
124063 / mocos
124134 / mprip
124240 / vkind
124637 / dhx9
124709 / nek8
124989 / cxxc1
125154 / zc3h4
125196 / ptpr2
125219 / kbtb2
125520 / foxn4
125661 / nvl
126364 / lar
126580 / ep300
126789 / est2e
126921 / rs9
126982 / gfpl
128224 / spt6h
128281 / lipa1
128562 / tep1
128687 / 
128832 / hells
129053 / ubc12
130062 / 
131119 / 
131216 / smc6
131808 / 
132176 / 
132295 / kapca
133792 / huwe1
133981 / kif19
135736 / ttc14
143664 / mdn1
146334 / zn268
153828 / sbno1
153994 / apc
183747 / lrp4
183809 / cl16a
201006 / kif15
201044 / rpa1
201563 / int6a
202549 / smag1
202569 / bub1b
202950 / wdfy3
204870 / nek1
205877 / yc006
206850 / rasa1
208117 / afad
208397 / aa2br
209728 / ddx3y
210487 / mabp1
215173 / nuak1
215827 / af10
218963 / chd7
219199 / ary1
220146 / myo16
221347 / mylk
223882 / taf11
225211 / dph2
227655 / thsd4
231202 / lrp4
232191 / dapk1
236233 / neur1
238365 / mgdp1
239931 / st38l
242546 / dyh12
243011 / hdgr3
245341 / smad4
266302 / ank1
274292 / srp09
281321 / rprd2
281429 / morc2
281464 / phip
283756 / msh6
285685 / rm45
287729 / rad50
Find  
Clear
  
  
 
  
  

 information 

1. JGI ID :
2. symbol :
3. description :
4. FPKM :
5. FC(log2) -
6. X:
     
   Y:
