# Supplementary material for: Molecular asymmetry in the cephalochordate embryo revealed by single-blastomere transcriptome profiling
Source: PLoS Genet. 2020 Dec 31;16(12):e1009294. doi: 10.1371/journal.pgen.1009294 (PMC7806126; doi:10.1371/journal.pgen.1009294)
Supplement: Attachment — Submitted filename: Lin_ResponsesToReviewers.docx [file pgen.1009294.s014.docx]

**Point-by-point responses to reviewers’ comments**

Reviewer #1: This manuscript describes single cell RNA-seq results with the aim to list the localized maternal mRNAs in cephalochordate embryos. The authors sequenced cDNA of isolated blastomeres of the 2-cell embryos and cDNA of separated animal and vegetal hemispheres at the 8-cell stage in Branchyostoma. The data was analyzed in pairwise way. Branchyostoma embryo has a single cluster of germ plasm that is partitioned into one of 2 cells at the 2-cell stage and then into the vegetal half at the 8-cell stage. The data suggests that 106 RNAs are localized to the germ plasm, and 222 RNAs to the animal hemisphere. They confirm this by in situ hybridization of 13 randomly selected germ plasm RNAs and 8 animal hemisphere RNAs. Germ plasm RNAs showed localization to tiny germ plasm, while animal hemisphere RNAs had rather broad distribution biased to the animal hemisphere. The author carried out GO analysis and functional analysis of the four animal hemisphere RNAs by overexpression in zebrafish embryos. Zfp665 and soxB1b resulted in dorsalization phenotype. Overall the data is clean and well presented. I appreciate comprehensive listing of localized maternal RNAs to the germ plasm in this study. However, there are some issues need to be addressed in the current manuscript.

Major comments:
1. Introduction, first paragraph. The authors mention that maternally localized cytoplasmic determinants encode transcription factors to provide “mosaic” properties for the blastomeres that inherit them, or alternatively, encode signaling molecules to generate “regulative” inductive signals. This sentence is misleading. Mosaic properties are not only accounted by localized transcription factors. It should be related to localization of maternal factors that are unrelated to the discrimination between transcription factors and signaling molecules. So it is inappropriate to relate localized signaling molecules to regulative property.

*- As suggested by reviewer #1 and #3, we have modified the sentences and added a reference:*

*“Modern molecular analyses have demonstrated that maternally localized cytoplasmic determinants confer “mosaic” properties to the blastomeres that inherit them, while cell-cell interactions via extracellular signals result in “regulative” development [2]. These cytoplasmic determinants and signaling pathways then activate downstream genes to specify different cell fates and embryonic polarities.”*

2. Introduction, second paragraph. The authors should read the following paper and update the descriptions written in this paragraph.
Maternal Huluwa Dictates the Embryonic Body Axis Through β-Catenin in Vertebrates
Lu Yan, Jing Chen, Xuechen Zhu, Jiawei Sun, Xiaotong Wu, Weimin Shen, Weiying Zhang, Qinghua Tao, Anming Meng
Science. 2018 Nov 23;362
doi: 10.1126/science.aat1045.

*- We have updated the information and cited the suggested reference:*

*“In addition to wnt ligands, Hwa, a maternal protein localized on the plasma membrane of dorsal blastomeres, protects β-catenin from degradation and is an essential determinant for the formation of the dorsal organizer in zebrafish and Xenopus embryos [6].”*

3. Introduction, third paragraph. The authors may state that cell fates in the animal hemisphere (ectodermal fates) are basic fates without localized determinant, and the vegetal cell fates were modified by the localized maternal factors in tunicates. In ascidians, formation of the animal-vegetal axis and the anterior-posterior axis is totally independent processes.

*- We have modified the last sentence of this paragraph into:*

*“Thus, these systematic studies suggest that transcriptional determinants of the early body plan in tunicate embryos are at least mostly localized to the posterior vegetal region to promote the posterior fate, while the lack of posterior-vegetal determinants in the anterior blastomeres results in default ectodermal specification [11].”*

4. Line 109. It is better to describe the results of the references 12 and 13 in more detail because these references are hardly obtained (downloaded).

*- As suggested by the reviewer, we have described parts of the published results in more detail in this paragraph (see line110-121).*

5. Line 114. amphioxus blastomeres separated at the 2-cell stage have different developmental capacities, suggesting that the cells are not identical and certain asymmetrically localized maternal transcripts likely participate in specifying cell fates and body axes in early amphioxus embryos [17].
I do not agree with this sentence. In their previous research (reference 17), the autors showed that blastomeres separated at the 2-cell stage develop into twin larvae that have normal morphology. Therefore, localized germ plasm is likely contribute to germ cell formation, but not to specifying cell fates and body axes in amphioxus embryos that show regulative property as reported since many years ago.

*- In our previous publication (Wu et al., 2011), we showed that amphioxus blastomeres separated at the 2-cell stage have different developmental capacities when raised to the larval stage. In most recorded cases (5 out of 6 pairs), one blastomere develops into a typical larva and the other one displays a curly-tail phenotype with disorganized posterior structures. Similar experiments were also carried out by Wilson (1893), Conklin (1933), and Tung et al. (1958). They did mention that certain amount of larvae derived from isolated blastomeres of the two-cell stage embryos had abnormal tails. However, these and later literatures tend to overlook this phenomenon and made the general conclusion that amphioxus blastomeres isolated at two-cell stage can develop into typical embryos and larvae. In Wu et al., 2011, we repeated these classic experiments with modern molecular markers, which enabled us to suggest that the curly-tail phenotype in those larvae is likely the result from lacking vegetal pole plasm in one of the first two blastomeres. In the revised manuscript, we elaborated the sentence as below:*

*“As such, amphioxus blastomeres separated at the 2-cell stage have different developmental capacities, with one blastomere developing into a typical larva and the other one becoming a larva with a curly-tail phenotype with disorganized posterior structures; thus, the first two blastomeres are not identical, and certain asymmetrically localized maternal transcripts likely participate in specifying cell fates and body axes in early amphioxus embryos [19].”*

6. Line 251. Due to the limitation of the detection method. Is there a possibility that the RNAs are gradually concentrated to germ plasm from the 2- to 8-cell stage?

*- We thank the reviewer for pointing out this possibility. We have added explanation as following and discussed another possibility that zygotic activation may also contribute to this phenomenon.*

*“In five cases (Fig 4Ca-c and 4Dj-k), the compact signal could not be detected until the 8-cell stage, possibly due to a limitation of the chromogenic detection method; the aggregated signal could be observed for two of the five transcripts at the 2-cell stage when fluorescence in situ hybridization was used (Fig 4Dj-k). It is also possible that some maternal transcripts may gradually become concentrated in the germ granule during the period from the 2- to 8-cell stage, or alternatively, zygotic gene activation between the 2- to 8-cell stage may generate transcripts that become associated with the granule (see further analysis below).”*

*- As suggested by Reviewer 2, we conducted additional analysis by comparing the 2-cell and 8-cell datasets. We have added these new results in Fig S8 and in a new result section “Differential expression analysis between the 2- and 8-cell stages”.*

7. Fig. 4Ci. FoxN transcripts seems to be localized to the vegetal hemisphere in addition to strong localization to the germ plasm. If this is reproducible result, the authors might mention about this.

*- Indeed, foxn1/4b and several other transcripts displayed visible cytoplasmic distribution in addition to the localization to the germ granule, and foxn1/4b transcripts were localized to the vegetal hemisphere as the reviewer pointed out. We have described these observations in the following sentences:*

*“In addition to the signals in the germ granule, transcripts of several genes that we examined displayed visible cytoplasmic distributions in diffused patterns (Fig 4Cc, 4Cf-i, 4Dm), similar to our previous observation for amphioxus vasa transcripts [19]. Furthermore, we observed foxn1/4b transcripts were asymmetrically localized to the vegetal hemisphere at the 2-cell stage and were more abundant in the germ granule-bearing blastomere at the 8-cell stage (Fig 4Ci).”*

8. Fig. 5C. I am wondering how the authors recognized the animal and vegetal poles of the two-cell stage embryos? This should be clearly mentioned.

*- We recognized the animal pole by the position of the polar body, which could be seen by DNA staining with Hoechst. We have provided a new supplementary figure (S6 Fig) and modified the sentence:*

*“We successfully detected signals for 10 of these transcripts (Fig 5A and 5B), and showed that nine (except naa38/279155) were clearly more concentrated toward the animal pole at the 2-cell stage (Fig 5C); at this stage, the animal pole could be recognized by the position of the polar body (S6 Fig).”*

Minor comments:
1. Titles of S1 Data Table. Titles corresponding to Fig. 2H-J are all “transcript list enriched in the germ granule-positive blastomere”. This would be mistake.

*- We thank the reviewer for pointing out this mistake. We have corrected those titles of S1 Data Table.*

2. Line 163. Interestingly, ratios of the reads mapped to the mitochondrial DNA did not show batch effects. I cannot understand this sentence. Be more specific.

*- To clarify our descriptions, we have modified the sentences:*

*“The proportion of reads mapped to mitochondrial DNA varied among samples, but this variation could not be attributed to their batch origins. Interestingly, we noticed that the proportion of reads mapped to mitochondrial DNA were slightly but consistently higher in germ granule-positive samples from both 2- and 8-cell stage embryo sets (paired t-test, p = 0.0064), indicating that more mitochondrial transcripts were produced in the germ granule-positive blastomeres.”*

3. Fig. S4. Readers would want to know the positions of the genes that is shown in Fig. 3B, namely, Tcf, nodal, SoxB1b etc.

*- We have added new panels in the revised Fig. S4 to indicate the positions of those genes.*

4. Fig. 4Ba. Syptonemal would be Synaptonemal.

*- We have corrected the spelling in the revised Fig. 4B. Please note that we have reordered the gene list and Synaptonemal complex protein 3 is now in Fig. 4Bg.*

5. Do the authors know the distribution of SoxB1b mRNA in zebrafish embryos?

*- We have added additional information in the Discussion section (line 549-555).*

Reviewer #2: In this study the authors apply RNAseq technology to examine the cellular transcriptomes of each cell of the 2-cell embryo and pooled cells of animal or vegetal identity from the 8 cell stage embryo to address the question of whether or not there is prepattern in this amphioxus species, which is thought to develop in a regulative manner. The authors identified markers that were uniquely expressed in one of two cells at the two stage and that showed differential expression between animal and vegetal halves of the embryo at the 8 cell stage. They went on to validate the expression of a subset of these markers in amphioxus embryos and found evidence that not all blastomeres are equivalent in this embryo. They went on to examine whether germ granule markers showed conserved localization to the germ plasm of zebrafish embryos, and performed overexpression analysis of putative patterning molecules to investigate their effects on zebrafish development. Overall the data are clearly presented, and the manuscript is well-written. The authors identified some new germ granule localized markers and differentially expressed genes that should pave the way for future functional analyses. However, there are a few points detailed below that should be addressed.

Major
When is the zygotic genome activated (ZGA)? Some of the transcripts do not appear to be present or not in the granule at in 2 cell stage, but are in the granule at 8 cell stage (Fig. 4). Similarly, Dag is animal, which seems counter intuitive for a granule marker, and in germ granule? Is this enrichment or de novo transcription in the granule? These should be discussed in the context of ZGA, which likely initiates by this time based on earlier work in other amphioxus species.

*- From published literatures, it was reported that zygotic gene expression could be observed as early as 64- to 128-cell stage (such as the lefty gene in Onai et al., 2010), which represents the very early blastula stage in amphioxus. To address the question raised by reviewers #2 and #3, we performed additional analysis by comparing the 2-cell and 8-cell datasets. We found that the levels of 1406 transcripts were significantly increased from 2-cell to 8-cell stage, suggesting that these genes are zygotically activated between these two stages. Therefore, ZGA in amphioxus likely occurs much earlier than previously thought. We have added these new results in Fig S8 and in a new result section “Differential expression analysis between the 2- and 8-cell stages” (line 421-447) and discussed in line 523-527.*

Related to this point, for the 8 cell stage experiments it appears two pools were created corresponding to animal and vegetal blastomeres. While this seems to be effective for distinguishing between animal and vegetal transcript differences, it assumes that all animal and all vegetal blastomeres are equivalent, which is not the case at least for vegetal since one vegetal blastomere is known to harbor the germ granule/aggregate. It is likely that this could/did result in dilution or underrepresentation of transcripts that may be uniquely enriched in the prospective germline cell or other cell types, especially since the PCR amplification likely introduces bias against low abundance transcripts. Nonetheless new granule localized markers were identified.

*- Indeed, distributions of transcripts may not be homogenous in each of the blastomeres within the animal and vegetal tiers of the 8-cell stage embryo. Thus, validation by WMISH is essential to reveal distributions of the DETs identified by our RNA-seq analyses. We have included additional explanation in the Discussion section (line 483-493).*

The authors perform comparative biological analysis by examining the expression patterns of granule enriched markers and potential axis markers in zebrafish. Among the markers examined for germline localization only one, vasa, was localized to the germ plasm of zebrafish. Based on this the authors conclude that germ granule transcripts are unique between species. However, only early stages (prior to genome activation) were examined. Related to the point above, if some of the markers that localized to the granule of 8 cell stage in amphioxus are zygotic products, then they might also only be localized/expressed/enriched in germ cells of zebrafish after genome activation, which occurs at a later stage than examined in this study. It is important to examine comparable stages when determining whether or not transcripts are unique to the germline in this case.

*- We have examined expression of genes at a later stage (30 hpf) after the primordial germ cells have migrated to the developing gonad. We found that, except vasa, 6 orthologs of the amphioxus germ granule-enriched transcripts that we tested were not detected in the gonad. We have added the new data in Fig. S5B and included in the result section (line 289-296).*

Are these genes localized to the germ granule of other amphioxus species?

*- Our previous publication (Zhang et al., EvoDevo 4:17, 2013) has described that maternal transcripts of vasa, nanos, piwil1, and tdrd7 in two Asian amphioxus, B. belcheri and B. japonicum, are localized in the germ granule, similar to the patterns seen in B. floridae. Dailey et al. (2016) also showed that vasa, nanos, piwil1, pl10, and bruno2 transcripts are localized to the germ granule in the European amphioxus B. lanceolatum. These results suggest that a core set of germline determinants is conserved across species. We have added this information in the result section (line 249-252). Further studies examining whether the newly identified germ granule-enriched transcripts show the same pattern in other amphioxus species are ongoing, and we plan to discuss these results in the context of evolution in a separate publication.*

No numbers are reported for the number of embryos examined and fraction with the pattern shown for the validation of candidates in Figs 4 and 5.

*- We have added the information in the figure legends of Fig. 4 and Fig. 5.*

It is unclear why the authors did not attempt to over express or target these genes in amphioxus to determine the developmental consequences. I did not find the overexpression analysis performed in the zebrafish added much to this study or to mechanisms regulating axis patterning. These experiments simply show that proteins that these proteins can dorsalize zebrafish embryos when overexpressed, which was already predictable based on their molecular identity and activities of proteins with similar functional domains in zebrafish. One limitation with these experiments is that there does not appear to be a corresponding zebrafish ortholog expressed at this time so one cannot perform complementary loss of function experiments to ask if the gene activity is required for patterning or not in zebrafish or experiments to determine if the amphioxus protein can rescue loss of the zebrafish protein. Moreover, it doesn’t really address the function of these genes in amphioxus development.

*- We agree with the reviewer that functional studies in amphioxus embryos are important and we understand the limitation of the zebrafish experiments presented here in this manuscript. We did attempt to conduct functional studies by knocking down zfp665 using a membrane permeable antisense vivo-morpholino (vMO) in the amphioxus embryos. As the results presented below, although zfp665 vMO affected axial development, we observed similar phenotype in the control embryos treated with a standard control vMO. We thus are not confident to include this data in the manuscript. During the revision process, we also tried to conduct overexpression experiment by injecting zfp665 mRNA into amphioxus eggs. Unfortunately, during the past months, we could not obtained mature gametes separately from the animals in our lab aquariums to perform such experiments. Due to the travel restriction caused by COVID-19 pandemic, we could not replenish our lab stock with wild animals. We are currently initiating a collaboration with other labs to conduct systematic functional studies on the newly identified maternal transcripts, including zfp665 and others that show asymmetrical localization in the early amphioxus embryo. We will continue this line of the research in the next few years, and results from these experiments should provide a more comprehensive understanding on their roles in amphioxus embryogenesis.*

For some of the RNAs the authors performed dose response curves, but in others the numbers seem rather low and there is no indication of how many times the experiments were repeated or how much variation there was from one experimental repetition to the next. This information and statistical analysis should be included. In addition for some experiments, for example the zfp665 experiments in Fig. 8 D, the numbers examined are rather low.

- *We have performed additional experiments to increase the sample sizes. All the overexpression experiments were performed in at least two different batches, and the results were consistent among batches. We have included this information and statistical analysis in the supplementary data (S1 Data) and presented in the new Figs. 7-9.*

Minor

In figure 7 and 8, what was the control for the injection experiments presumably gfp was the control, but the labels just say “control”? If so, please label as gfp and with the concentration injected rather than just control.

*- Indeed, these control embryos were injected with gfp mRNA. We have changed the labels from “control” to “gfp”.*

How was the RNA precipitated? This should be indicated in the methods section.

*- The RNAs were purified by passing through a MEGAclear column (Life Technologies) and precipitated with ammonium acetate and ethanol. We have added this information in the Methods section (line 730-732).*

Measuring the angle of the expression domain has become common practice for quantifying expansion or reduction of DV patterning marker expression and should be included.

*- We have measured the angle of the expression domains to quantify the effects. The results are included in the new Fig. 8 and described in the text (line 397-401).*

The numbers (fraction similarly affected) are reported on some of the figure panels but not on others. This should be shown consistently throughout the manuscript.

*- We have modified the presentations in the new Fig. 8 and provided quantitative measurements in a more uniform format. For bmp2b, because its expression could be detected in both the ventral and dorsal side (stronger on the ventral side), similar quantitative measurement could not be applied. We thus presented the bmp2b images in the lateral view with numbers.*

Presumably Figure S4 color code is the same as in Fig. 3 but a key should be included in the figure.

*- We have added the color codes in Fig. S4.*

Reviewer #3: In this manuscript Lin and collaborators describe, using a single cell/low cell number RNAseq approach the transcriptomic asymmetry in amphioxus cells at two early developmental stages, the two cell and eight cell stages. The study is correctly performed and the results generated may be very useful for the amphioxus community but also for EvoDevo researchers.
The generated transcriptomic data allowed to identify several hundreds of differentially expressed transcripts (DET) at these two developmental stages, between the two cells (in the 2cell stage) and between the micromers and macromers (in the 8 cell stage). The authors confirmed their results using in situ hybridization of some of the DET and they tested functionally by overexpression of two of them (as well as chimeric constructions of one of them, zpf665) in zebrafish, showing its capacity to dorsalize the zebrafish embryo.
The manuscript is correctly written, it is clear and interesting, but have some comments/criticisms that should be corrected/added before publication. So my advice is “major corrections”.
Comments/criticisms:
- Lines 66-70: “Modern molecular analyses have demonstrated that maternally localized cytoplasmic determinants encode transcription factors to provide “mosaic” properties for the blastomeres that inherit them, or alternatively, encode signaling molecules to generate “regulative” inductive signals that instruct nearby cells.” Please add references

*- As suggested by reviewer #1 and #3, we have modified the sentences and added a reference.*

*“Modern molecular analyses have demonstrated that maternally localized cytoplasmic determinants confer “mosaic” properties to the blastomeres that inherit them, while cell-cell interactions via extracellular signals result in “regulative” development [2]. These cytoplasmic determinants and signaling pathways then activate downstream genes to specify different cell fates and embryonic polarities.”*

- Lines 173-174: “a total of 23,517 genes had detectable transcript levels”. In the B. floridae genome paper (Putnam et al 2008), the authors found 21,900 coding genes. Please explain how did you find 23517 genes with detectable transcript levels

*- We used the updated version of the Branchiostoma floridae genome (JGI v2.0) containing 28666 annotated gene models. The version of the genome and its source were included in the Methods section (line 654-656).*

-While the authors compare the transcriptome between the two cells of the 2cell stage, or the micromers versus macromers in the 8c stage, I suggest to also compare the 2cell stage with the 8cell stage transcriptomes. Is there any transcription between these two early developmental stages? If any, is this transcription also asymmetric?

*- As suggested by reviewers #2 and #3, we performed additional analysis by comparing the 2-cell and 8-cell datasets. We found that the levels of 1406 transcripts were significantly increased from 2-cell to 8-cell stage, suggesting that these genes are zygotically activated between these two stages. Among those 1406 transcripts, 19 were differentially enriched either in the animal (14 transcripts) or vegetal tier (5 transcripts) at the 8-cell stage. We have added these new results in Fig S8 and in a new result section “Differential expression analysis between the 2- and 8-cell stages” (line 421-447) and discussed in line 523-527.*

-I find that the DETs enriched in the germ-granule negative blastomer is very low, and particularly the number of DET transcripts shared between the two methods used. Is there any explanation for that?

*- We think these results suggest that the presence of the germ granule is the predominant factor contributing to the molecular asymmetry at the two cell stage. The number of DETs enriched in the germ granule-negative blastomere identified by both methods is particularly low. This is because most of these DETs are at low levels and tend to be removed by the DESeq2 method. We have added this possible explanation in line 236-239.*

-The authors selected several genes for in situ verification of the RNAseq results but they do not detail how did they chose these genes. Why these genes and not others? Why only 13? 13 doesn’t seem a random number. Does it mean that other genes were also used for in situ but the in situ didn’t work? And finally why the authors only performed double in situ with four of them?

*- For the current manuscript, we selected 23 transcripts that are enriched in both the germ granule-positive blastomere and the vegetal tier, and 10 transcripts enriched in either the germ granule-positive blastomere or the vegetal tier for WMISH. The decision to select these genes is because we could readily obtain these cDNA clones from our existing EST library. Among the 33 WMISH analyses, we successfully detected signals for 13 of these transcripts. For the rest 20 genes, we could only detect background-like signals. We choose 4 (out of the 13) genes to do double in situ; among these four, when the chromogenic in situ was performed, two show clear aggregation at both the 2- and 8-cell stages, while in the other two the compact signals were not detected at the 2-cell stage but the signals showed up at the 8-cell stage. We have added additional text to explain this in the revised manuscript (line 261-267). Further thorough analyses on all the asymmetrically distributed transcripts are ongoing in our laboratory.*

-In the case of the in situs performed with genes expressed in the animal tier (germ granule negative) blastomers, the authors did not explain why they chose 10 and not 13 as in the previous case, and again why did they chose these 10.

*- Similar to our previous reply, from our EST library, we obtained 22 cDNA clones of the animal tier-enriched transcripts for WMISH. We successfully detected signals for 10 of these transcripts and presented in Fig. 5C. We have added additional text to explain this in the revised manuscript (line 298-302).*

- Please exchange the order of phrases in lines 250-251 (“In five cases (Fig 4Ce-g and 4Dk-l), the compact signal could not be detected until the 8- cell stage, possibly due to the limitation of the detection method”) and lines 247-249.

*- We have exchanged the order of the phrases as suggested by the reviewer; we also expanded the text in this paragraph to incorporate comments from reviewers #1 and #2 (line 267-281).*

-Concerning the functional approaches, I understand that functional experiments in amphioxus are technically challenging, but other laboratories publish overexpression of RNAs, morpholinos, and even knock-out lineages. I understand that knockout is a really difficult and long experiment, but overexpression of the two mRNAs used in zebrafish (and the chimeras) should be possible in amphioxus isn’t it? This would show whether the phenotype observed in zebrafish is conserved.

- *We indeed tried to conduct overexpression experiment by injecting mRNAs into amphioxus eggs. Unfortunately, during the past months, we could not obtained mature gametes separately from the animals in our lab aquariums to perform such experiments. Due to the travel restriction caused by COVID-19 pandemic, we could not replenish our lab stock with wild animals. We are currently initiating a collaboration with other labs to conduct systematic functional studies on the newly identified maternal transcripts, including zfp665 and others that show asymmetrical localization in the early amphioxus embryo. We will continue this line of the research in the next few years, and results from these experiments should provide a more comprehensive understanding on their roles in amphioxus embryogenesis.*

-Concerning the phenotype of the dorsalized zebrafish embryos (both in figure 7 A and C and figure 8D), it would be useful to add some in situs with marker genes showing such dorsalization. I believe the authors when they say that these animals are dorsalized, but it is much better to show it clearly with in situs.

*- We have performed in situ hybridization on the genes that mark dorsally derived tissues, including pax2.1 (marking midbrain-hindbrain boundary), krox20 (labeling rhombomeres 3 and 5), myoD (dorsal mesoderm), and six3a (forebrain) at the 10 somite stage zebrafish embryos. The results support the dorsalization phenotype and we have included the data in the new Fig. 7 and Fig. 9. These new data have been incorporated into the Results section (line 367-374 and line 410-416).*
