# Supplementary material for: Molecular asymmetry in the cephalochordate embryo revealed by single-blastomere transcriptome profiling
Source: PLoS Genet. 2020 Dec 31;16(12):e1009294. doi: 10.1371/journal.pgen.1009294 (PMC7806126; doi:10.1371/journal.pgen.1009294)
Supplement: Attachment — Submitted filename: ResponsesToReviewers_2nd.docx [file pgen.1009294.s015.docx]

Reviewer's Responses to Questions

**Comments to the Authors:
Please note here if the review is uploaded as an attachment.**

Reviewer #1: The authors have responded thoroughly and satisfactorily to my concerns and most of the others comments on the earlier submission. The currently submission is greatly improve over the original submission.
I have three trivial comments.
1. line 102. The sentence would be “A lack of strong anterior determinants would be consistent with the observation that removal of the anterior cytoplasm has no effect on tunicate embryogenesis [10].”

- We have corrected the sentence as suggested by the reviewer.

2. line 106. The sentence would be “while the lack of posterior-vegetal determinants in the anterior blastomeres results in default anterior cell fate specification [11]." Please note that the anterior-posterior axis is defined perpendicular to the animal-vegetal axis in ascidian embryos. There should be vegetal hemisphere determinant in ascidian eggs, but the identity of it is still elusive. Presence of animal hemisphere determinants is also unclear. So far the posterior-vegetal determinants have been identified.

- We thank the reviewer for pointing out this. We have modified the text as suggested.

3. line 118. flatted should be replaced with flattened.

- The typo has been corrected.

Reviewer #2: This is a revised manuscript reporting results from RNAseq studies to examine the cellular transcriptomes of each cell of the 2-cell embryo and pooled cells of animal or vegetal identity from the 8 cell stage embryo to address the question of whether or not there is prepattern in this amphioxus species, which is thought to develop in a regulative manner. The authors identified markers that were uniquely expressed in one of two cells at the two stage and that showed differential expression between animal and vegetal halves of the embryo at the 8 cell stage. They went on to validate the expression of a subset of these markers in amphioxus embryos and found evidence that not all blastomeres are equivalent in this embryo. To explore potential conserved gene functions, they examined whether germ granule markers showed conserved localization to the germ plasm of zebrafish embryos, and performed overexpression assays to investigate the function/activity of putative patterning molecules on zebrafish development. Overall the data are clearly presented, and the manuscript is well-written. The authors identified some new germ granule localized markers and differentially expressed genes that should pave the way for future functional analyses. In this revision they also report evidence suggesting that zygotic transcription begins earlier than previously thought. In this version the authors have addressed my previous concerns; however, a few points remain to be addressed or clarified.

1) The authors report that zygotic transcription begins much earlier than previously expected. This is an interesting and important addition to the manuscript. Because early development, prior to genome activation, is regulated post-transcriptionally, the authors should indicate how they distinguished between de novo transcription and changes in polyadenylation.

- We thank the reviewer for bringing out this point. Our presented analyses are based on polyadenylated transcripts. With the current data, we are unable to distinguish between early zygotic activation and changes in polyadenylation. We have added the following text in Discussion to discuss this possibility:

*It should be noted that our analyses were based on measurements of polyadenylated transcripts and did not include transcripts without polyadenylation. It was previously shown that specific maternal mRNAs in Xenopus are polyadenylated or deadenylated after fertilization (Graindorge et al., 2006). RNA-seq analyses in the marine annelid Platynereis dumerilii also suggest that polyadenylation may partly contribute to changes in developmental expression profiles shortly after fertilization (Nakama et al., 2017). Thus, it is possible that some of the “early activated genes” we identified are actually maternally loaded transcripts that are polyadenylated between the 2- to the 8-cell stage. Additional RNA-seq experiments using mRNAs captured by different methods (e.g., Blower et al., 2013) would be required to further address this issue.*

2) I understand that the functional studies using amphioxus embryos are challenging and appreciate the authors efforts to attempt to assess gene function in amphioxus. I also understand that because this proved difficult the authors had to rely on functional assays using zebrafish embryos to explore potential activities of their candidate patterning genes. To better distinguish between actual requirements/roles in patterning in this case and potential gene functions or activities, which is what the authors really show, it seems more appropriate to replace “regulates” in the section titled “Amphioxus zfp665 represses..”with “disrupts” or something similar. e.g. (pg. 17 line 395) “to further understand how amphioxus zfp665 regulates axial patterning…” would become “to further understand how amphioxus zfp665 disrupts axial patterning…” and later (pg. 17 line 402) “suggest that zfp665 regulates/…” would become instead “suggest that zfp665 disrupts or perturbs…..” (pg. 18 line 418) “to regulate zebrafish dorsoventral patterning” would be “to disrupt or interfere with zebrafish dorsoventral patterning”

- We thank the reviewer for the suggestion and have modified the sentences accordingly.

Reviewer #3: Most of my comments and criticisms have been answered correctly. In addition, the authors have added some analyses on the RNAseq samples I requested and the supplementary results are of great interest.
For these reasons, and despite not having been able to perform the over-expression experiments on amphioxus embryos, for reasons I can understand, I accept the manuscript for publication in PloS Genetics

- We thank the reviewer for the comments.
